# Molecular mechanisms affected by boron deficiency in root and shoot meristems of plants

**DOI:** 10.1093/jxb/eraf036

**Published:** 2025-01-28

**Authors:** Liuyang Chu, Cay Christin Schäfer, Michaela S Matthes

**Affiliations:** University of Bonn, Institute for Crop Science and Resource Conservation, Crop Functional Genomics, Friedrich-Ebert-Allee 144, 53113 Bonn, Germany; University of Bonn, Institute for Crop Science and Resource Conservation, Crop Functional Genomics, Friedrich-Ebert-Allee 144, 53113 Bonn, Germany; University of Bonn, Institute for Crop Science and Resource Conservation, Crop Functional Genomics, Friedrich-Ebert-Allee 144, 53113 Bonn, Germany; University of Maryland, USA

**Keywords:** Auxin, boron deficiency, cytokinin, ethylene, meristem

## Abstract

Boron deficiency is an abiotic stress that negatively impacts plant growth and yield worldwide. Boron deficiency primarily affects the development of plant meristems— stem cells critical for all post-embryonic tissue growth. The essential role of boron in meristem development was first established in 1923. It remains unclear whether boron directly integrates into meristem molecular signalling pathways. In addition to its stabilizing function in the primary cell wall, growing evidence suggests roles for boron in various molecular processes including phytohormone cascades. These indications enhance a mechanistic understanding of why boron is crucial for proper meristem development. In this review we compile and discuss molecular pathways influenced by boron availability in Arabidopsis (*Arabidopsis thaliana*), maize (*Zea mays*), rice (*Oryza sativa*), and oilseed rape (*Brassica napus*) with a focus on the auxin-, ethylene-, and cytokinin-mediated hormone cascades. We particularly compare and contrast phenotypic and molecular adaptations of shoot and root meristems to boron deficiency and pinpoint tissue-specific differences.

## Teaser

The primary objectives of this review are to compile, summarize, and link molecular processes affected by boron deficiency to the phenotypic defects observed in plant meristems with a focus on potential boron-hormone interactions.

## Introduction

Deficiency of the micronutrient boron in soils is a prominent abiotic stress worldwide which negatively affects various developmental processes in plants (as reviewed in Matthes *et al.*, 2020; Bolaños *et al.*, 2023). This is seen from the phenotypes arising under boron-deficient conditions, which mainly affect the growing tips of plant roots and shoots. Both halt their growth under such conditions, leading to the inhibition of root elongation, leaf expansion, and flower development. Reproductive development is particularly sensitive to boron deficiency. For example, in the cereal crop maize (*Zea mays* L.) both male and female inflorescences are completely missing under severe boron deficiency (Durbak *et al.*, 2014), while in less severe cases of boron deficiency, pollen sterility and small or deformed cobs are observed (Lordkaew *et al.*, 2011). The severe defects during reproductive development consequently lead to drastic yield losses when soil boron availability is limited (as reviewed in Brdar-Jokanović, 2020). The sensitivity of reproductive development to boron deficiency is not specific to maize, but is also observed in many other crops, including rice (*Oryza sativa*) and oilseed rape (*Brassica napus*).

All boron deficiency-induced phenotypes can be linked to defects during shoot and root meristem development. Meristems are groups of stem cells that are responsible for all post-embryonic organ development in plants and are subdivided into zones, which are defined by their function and position (Barton, 2010). These zones are necessary for maintaining a meristematic state and for the initiation of organ primordia. The tight balance between stem cell maintenance and differentiation processes in meristems leads to proper plant growth. Genetic factors balancing differentiation and maintenance in plant meristems revolve around multiple *CLAVATA-* (*CLV*) related signaling pathways, which converge onto the WUSCHEL (WUS) transcription factor (as reviewed in Somssich and Je, 2016). Parallel to the CLV-WUS pathways, meristem development is tightly linked to the action of various transcription factors and several classes of phytohormones. They include auxins, cytokinins, gibberellins, brassinosteroids, ethylene, and abscisic acid (as reviewed in Somssich and Je, 2016). The molecular mechanisms relating to how boron affects meristem development, however, are not resolved.

The only characterized function of boron is in the crosslinking of two pectic subunits of rhamnogalacturonan-II (RG-II) in the primary cell wall (Kobayashi *et al.*, 1996; Matoh *et al.*, 1996; O’Neill *et al.*, 1996), which contributes to its stability. It is also known that boron, in form of boric acid, diffuses into plant cells under boron-sufficient conditions (Raven, 1980; Dordas and Brown, 2000), and that facilitated transport of boron is needed to provide sufficient boron to plants if soil boron levels are low (as reviewed in Miwa and Fujiwara, 2010). The main boron import facilitator gene in the Arabidopsis (*Arabidopsis thaliana*) root is a member of the NODULIN INTRINSIC PROTEIN (NIP) family, namely NIP5;1 (Takano *et al.*, 2006). Orthologs have been identified in many major crops, including maize (*tassel-less1; tls1*) (Durbak *et al.*, 2014; Leonard *et al.*, 2014), rice (*DWARF AND TILLER-ENHANCING (DTE)*, *NIP3;1*, *APICAL SPIKELET ABORTION (ASA),* and *Low Silicon 1 (LSI1)*) (Hanaoka *et al.*, 2014; Liu *et al.*, 2015; Shao *et al.*, 2018; Zhou *et al.*, 2021), and oilseed rape (Hua *et al.*, 2016a; He *et al.*, 2021a; Song *et al.*, 2021). Boron levels inside plant cells are tightly regulated and boron, in form of the borate anion, is exported out of the cell via members of the HIGH BORON REQUIRING (BOR) family, with the main boron export protein in Arabidopsis being BOR1 in root tissues (Noguchi *et al.*, 1997). Likewise, orthologs of *BOR1* were identified in various crops including maize, rice, and oilseed rape (Nakagawa *et al.*, 2007; Chatterjee *et al.*, 2014, 2017; Zhang *et al.*, 2017). Due to impaired boron transport, boron transporter mutants are inherently boron-deficient and consequently show enhanced boron deficiency-related phenotypes (Noguchi *et al.*, 1997; Takano *et al.*, 2006; Chatterjee *et al.*, 2014, 2017; Durbak *et al.*, 2014; Hanaoka *et al.*, 2014; Leonard *et al.*, 2014; Liu *et al.*, 2015; Shao *et al.*, 2018; Song *et al.*, 2021; Zhou *et al.*, 2021).

Based on observations that defects in pectin crosslinking cannot explain all phenotypes observed under boron-deficient conditions and that there are striking differences of phenotypes observed in boron transporter mutants compared with wild-type plants grown under boron-deficient conditions (as reviewed in Matthes *et al.*, 2020), additional cellular functions of boron were suggested including roles in plasma membrane structure and function, or involvements in phytohormone-related processes (Rengel *et al.*, 2022). Since meristem developmental cascades are processes in which phytohormones play crucial signaling and regulatory roles, the potential involvement of boron in hormonal pathways are intriguing and might provide at least some explanations as to why meristem development is affected under boron-deficient conditions.

In this review we compile, discuss, and contrast the molecular processes that correlate with altered root or shoot meristem development under boron-deficient conditions. We highlight indications for interactions of boron with the phytohormones auxin, ethylene, and cytokinin in Arabidopsis, the cereal crops maize and rice, and the oilseed crop oilseed rape. We further point out involvements of boron in meristem development pathways revolving around the *CLV-WUS* and specific transcription factor-mediated pathways.

## Boron deficiency particularly affects plant meristems

Early boron research identified its unique role in inducing meristematic activity and a high degree of tissue-specificity, including both cell elongation and cell division defects (Warington, 1923; Sommer and Sorokin, 1928; Josten and Kutschera, 1999). The molecular basis underlying the boron deficiency-induced shoot and root meristem defects, however, remained widely unexplored. The sensitivity of meristems to boron deficiency is specific, as negative effects on meristematic tissues have only been reported for calcium, copper, iron, and zinc deficiencies (Rengel *et al.*, 2022).

### Boron deficiency-induced defects in root meristems

Boron deficiency in Arabidopsis, maize, rice, and oilseed rape leads to rapid defects in primary and/or lateral root elongation, causes complete absence of the apical meristems, and affects lateral root number (Eltinge, 1936; Josten and Kutschera, 1999; Takano *et al.*, 2006; Nakagawa *et al.*, 2007; Martín-Rejano *et al.*, 2011; Abreu *et al.*, 2014; Chatterjee *et al.*, 2014, 2017; Durbak *et al.*, 2014; Camacho-Cristóbal *et al.*, 2015; Hua *et al.*, 2016b; Li *et al.*, 2016; Poza-Viejo *et al.*, 2018; Housh *et al.*, 2020; He *et al.*, 2021b; Wilder *et al.*, 2022; Verwaaijen *et al.*, 2023). In addition, swollen and necrotic root tips with larger cells are characteristic for boron-deficient roots (Sommer and Sorokin, 1928; Abreu *et al.*, 2014; Camacho-Cristóbal *et al.*, 2015; Li *et al.*, 2016).

During Arabidopsis root development, early defects upon boron limitation include reduction of the meristematic zone and abnormal cell differentiation, with the severity of this reduction in meristem size increased with the degree of boron limitation (Abreu *et al.*, 2014). Likewise, the ability to revert the boron deficiency-induced root meristem defects by resupplying boron decreased with increasing severity of boron deficiency. These root meristem defects are causative for the reduction in primary root length under boron deficiency (Poza-Viejo *et al.*, 2018). A reduction of root meristem length was reported four hours post-transfer of seedlings from control to low boron conditions, whereas primary root length was inhibited starting 24 hours post-transfer (Poza-Viejo *et al.*, 2018). In addition, inhibition of cell division was an early response in the boron-deficient Arabidopsis root (Abreu *et al.*, 2014; Poza-Viejo *et al.*, 2018).

Studies regarding boron deficiency-induced root defects in crops are limited, mainly because of the restricted access of crop root systems grown under field conditions and the limitations of growing crops on agar plates. The reported root phenotypes, however, are indicative of meristem defects.

In maize, recent research suggests genotype- and tissue-specificity regarding the importance of boron for root development, with a low boron requirement for primary root development and a higher demand for lateral root development. The number of lateral roots was reduced under boron-deficient conditions (0 mM added boric acid) compared with sufficient (0.05 mM added boric acid) or excess boron conditions (0.5 mM added boric acid; Wilder *et al.*, 2022). Similar defects were observed using the putative boron deficiency mimic phenylboronic acid (PBA; Housh *et al.*, 2020). Maize seedlings that germinated from kernels exposed to PBA developed significantly shorter primary roots and lower lateral root density compared with seedlings from kernels germinated in ultra-pure water (Housh *et al.*, 2020). Furthermore, maize boron transporter mutants *tls1* and *rte2* showed shorter root systems compared with wild-type siblings under different boron conditions (*tls1*: boron-sufficient conditions; *rottenear2* (*rte2*): boron-deficient conditions) (Chatterjee *et al.*, 2014, 2017; Durbak *et al.*, 2014). This phenotype was enhanced in *rte;rte2* double mutants, which had significantly reduced primary root lengths and reduced lateral and seminal root numbers compared with wild-type siblings (Chatterjee *et al.*, 2017). No visible symptoms, however, were present in any part of wild-type maize at the five-leaf stage between boron-deficient and boron-sufficient conditions (Lordkaew *et al.*, 2011). Similarly, using a boron-deficient mineral substrate, different root traits were unaffected by boron deficiency in the wild type, despite boron deficiency symptoms appearing in leaf blades (Bienert *et al.*, 2023).

Likewise, in rice, no significant differences in root parameters were observed in young wild-type seedlings or in the boron import mutant *asa* under boron-deficient conditions (Uraguchi and Fujiwara, 2011; Zhou *et al.*, 2021). This is in contrast to the rice *bor1* export mutant, which showed reduced root growth under boron-deficient conditions (Nakagawa *et al.*, 2007).

Boron deficiency in oilseed rape caused immediate inhibition of primary and lateral root growth (Verwaaijen *et al.*, 2023). In addition, the density of root hairs and the root diameter increased in boron-deficient seedlings, while several other root traits, including total root length and root dry weight, decreased (Hua *et al.*, 2016b). The analysis of oilseed rape boron transporter mutants showed that mainly *Bn*A3.NIP5;1 promotes root elongation under low boron conditions (He *et al.*, 2021b).

### Boron deficiency-induced defects in shoot meristems

Boron deficiency-induced shoot phenotypes in Arabidopsis included reduced shoot growth, small and wrinkled leaves, loss of apical dominance, a delay of flower development, and reduced fertility (Noguchi *et al.*, 1997; Takano *et al.*, 2006). These defects were seen in the Arabidopsis boron transporter mutants *nip5;1* and *bor1-1* (Noguchi *et al.*, 1997; Takano *et al.*, 2006) and are all indicative of shoot apical meristem defects. Systematic analysis of these defects, however, is still pending.

In the maize boron transporter mutants, shoot meristem development is inhibited (Chatterjee *et al.*, 2014, 2017; Durbak *et al.*, 2014; Leonard *et al.*, 2014). Both the shoot apical meristem and the inflorescence meristems were shorter in height compared with wild-type controls (Chatterjee *et al.*, 2014, 2017; Durbak *et al.*, 2014). These phenotypes were shown to get progressively worse over time, suggesting that meristem maintenance is affected in these mutants. Indeed, double mutant analyses of *tls1* in combination with various mutants in meristem development pathways suggested functional involvement of boron or boron transport in specific meristem pathways (Matthes *et al.*, 2022). For example, mutation of *tls1* and the *CLV1* ortholog, *thick tassel dwarf1* (Bommert *et al.*, 2005), showed a partial rescue of the *tls1* tassel phenotype, whereas double mutants of *tls1* and the *CLV2* ortholog*, fasciated ear2* (Taguchi-Shiobara *et al.*, 2001) did not. In addition, double mutants of *tls1* and the meristem maintenance regulator *knotted1* (*kn1*) (Vollbrecht *et al.*, 1991) enhanced the boron-deficiency induced shoot and inflorescence meristem phenotypes of *tls1* single mutants (Matthes *et al.*, 2022), showing genetic interactions between *tls1* and *kn1*. These findings suggested a specific role of boron during meristem development and corroborated early studies in sunflower (*Helianthus annuus*; Josten and Kutschera,1999).

Reduced boron availability also impacts rice shoot phenotypes. Mutants in the rice *NIP3;1* boron transporter gene depicted vegetative and reproductive defects, including reductions in seed set and pollen viability (Liu *et al.*, 2015; Zhou *et al.*, 2021). These phenotypes were also observed in wild-type rice plants grown under prolonged boron limitation (Uraguchi and Fujiwara, 2011), suggesting boron deficiency conditions also impact the shoot meristem in rice. Indeed, the *lsi1* mutant is characterized by reduced growth of the leaf meristem when grown under boron-deficient conditions (Shao *et al.*, 2018). In addition, *asa* mutants display reduced fertility with defective spikelet meristem formation and severe reductions of panicle length and number. The apical spikelets were partly sterile and the pollen grains depicted an irregular morphology (Zhou *et al.*, 2021). Similarly, *bor1* mutants showed reduced shoot growth under boron-deficient conditions, but specific meristem phenotypes have not been investigated (Nakagawa *et al.*, 2007).

In wild-type oilseed rape, reproductive growth was more severely affected compared with vegetative growth under boron-deficient conditions (Song *et al.*, 2021; Verwaaijen *et al.*, 2023). Mutants in the orthologs of *bor1, bor2,* and *nip5;1*, however, showed severe boron deficiency defects during vegetative development, with phenotypes including stunted growth, dark green and malformed leaves, and inhibition of shoot apices (Zhang *et al.*, 2017; He *et al.*, 2021b; Liu *et al.*, 2024). Boron deficiency in oilseed rape additionally led to the abortion of flowers prior to seed setting and to a higher number of abnormal flowers, ultimately restricting seed yield (Hua *et al.*, 2016b; Zhang *et al.*, 2017; He *et al.*, 2021b; Song *et al.*, 2021; Verwaaijen *et al.*, 2023). The observed phenotypes were linked with reduced shoot apical meristem activity (Pommerrenig *et al.*, 2022) or were indicative of floral meristem defects. They have not been studied on a cellular level.

## Molecular mechanisms that correlate with the boron deficiency-induced meristem phenotypes

### Boron and meristem maintenance pathways

Boron research in recent years has made great progress in proposing molecular pathways leading to the observed phenotypic defects and to map out tissue specific differences. In root and shoot meristems, boron deficiency was shown to target meristem maintenance (Poza-Viejo *et al.*, 2018; Matthes *et al.*, 2022). In the Arabidopsis root meristem, boron deficiency primarily inhibited cell division and the boron deficiency-induced reduction of root meristem growth preceded the primary root elongation defects. The loss of quiescent center (QC) identity, where stem cells rarely divide, was subsequent to this inhibition, suggesting that the Arabidopsis root QC sustains growth at least to some extent under boron-deficient conditions (Poza-Viejo *et al.*, 2018).

The boron-deficient maize tassel meristem showed meristem maintenance defects alongside cell division defects (Chatterjee *et al.*, 2014, 2017; Durbak *et al.*, 2014; Matthes *et al.*, 2022). The detected molecular defects were shown to be specific, involving various transcription factors, genes involved in hormonal pathways, and cell division genes (Matthes *et al.*, 2022). A similar observation was made in shoot meristems of pea plants (*Pisum sativum*; Chen *et al.*, 2024). Boron deficiency-regulated transcription factors known to be involved in meristem development included the ETHYLENE RESPONSIVE ELEMENT BINDING/APETALA2 transcription factor family involved in the ethylene-hormone cascade (Matthes *et al.*, 2022; Verwaaijen *et al.*, 2023; Chen *et al.*, 2024) and specific homeobox transcription factors, like KN1 in maize (Matthes *et al.*, 2022) or HOMEOBOX PROTEIN KNOTTED1-LIKE 3/4 in pea (Chen *et al.*, 2024). In addition, a global ribosequencing experiment in Arabidopsis shoots detected boron-dependent gene regulation via ribosome stalling of several meristem development genes (Sotta *et al.*, 2021). These findings from various plant species suggest an involvement of boron in specific meristem pathways.

Furthermore, boron transporter genes show high expression particularly in meristematic tissues. The expression of these transporter genes was high in both the Arabidopsis root meristem (Sotta *et al.*, 2017) and in the maize tassel meristem (Matthes *et al.*, 2022), emphasizing the importance of boron and its active transport into meristematic tissues. In addition, the highest concentration of soluble boron was found around the QC in Arabidopsis and oilseed rape roots (Shimotohno *et al.*, 2015; He *et al.*, 2021a), which implies that boron is needed to maintain root apical meristem activity. This corroborated previous hypotheses that a threshold concentration of boron is needed in meristematic cells to promote division and subsequent expansion to ensure growth (Lovat, 1985).

### Indications for boron-hormone interactions

The pivotal role of phytohormone pathways during meristem development is undebatable. Boron deficiency leads to alterations in various phytohormone pathways, primarily the ethylene-, auxin-, and cytokinin-mediated cascades (Tables 1, 2; Supplementary Tables S1, S2). The mechanisms underlying putative boron-hormone interactions have not been resolved, but various observations support functional relationships. For example, various phytohormone levels were altered in boron-deficient compared with boron-sufficient plant tissue (as reviewed in Chen *et al.*, 2022; Matthes, 2022), and hormone mutants show altered growth under boron-deficient conditions (Martín-Rejano *et al.*, 2011; Camacho-Cristóbal *et al.*, 2015; Li *et al.*, 2015; Poza-Viejo *et al.*, 2018; Herrera-Rodríguez *et al.*, 2022; Matthes *et al.*, 2023; Tao *et al.*, 2023). Furthermore, transcripts of various hormone-related genes were differentially expressed under boron-deficient conditions (Zhou *et al.*, 2016; Matthes *et al.*, 2022; Tao *et al.*, 2023; Verwaaijen *et al.*, 2023), and the promoters of boron transporter genes harbor binding sites for hormone-related transcription factors (Gómez-Soto *et al.*, 2019; Matthes *et al.*, 2022).

**Table 1.** Molecular alterations in the auxin-mediated hormone cascade upon boron deficiency (-B) in *Arabidopsis thaliana* (*A. thaliana*), maize (*Z. mays*), and rice (*O. sativa*).

Auxin cascade	Species	Organ/Tissue	Conditions (-B)	Molecular component	Effect (-B)	Literature
Biosynthesis	*A. thaliana*	Shoot	Transfer assay, 50 µM B 4 d → 0 µM B (2d)	*TAA1, YUC3/9, NIT1*	Up-regulated	Tao *et al.* (2023)
Signaling	*A. thaliana*	Cotyledon, hypocotyl	Transfer assay, 50 µM B 4 d → 0 µM B (2d)	*DR5:GFP*	Increased	Tao *et al.* (2023)
Signaling	*A. thaliana*	Cotyledon, hypocotyl	Transfer assay, 50 µM B 4 d → 0 µM B (2d)	*DII-VENUS*	Decreased	Tao *et al.* (2023)
Transport	*A. thaliana*	Shoot	Transfer assay, 50 µM B 4 d → 0 µM B (2d)	*PIN2/3*	Up-regulated	Tao *et al.* (2023)
Transport	*A. thaliana*	Shoot	Transfer assay, 50 µM B 4 d → 0 µM B (2d)	*PIN1/4/7, AUX1*	Not altered	Tao *et al.* (2023)
Transport	*A. thaliana*	Cotyledon	Short term PBA-treatment (10 mM for 30 min)	PIN1-GFP	Altered localization	Matthes *et al.* (2016)
Auxin levels	*A. thaliana*	Whole root, root MZ, TZ, EZ	Transfer assay, 50 µM B 4 d → 0 µM B (2d)	IAA	Increased	Tao *et al.* (2023)
Biosynthesis	*A. thaliana*	Root	Transfer assay, 50 µM B 4 d → 0 µM B (2d)	*TAA1, YUC3/9, NIT1*	Not altered	Tao *et al.* (2023)
Signaling	*A. thaliana*	Root MZ, TZ, EZ	Transfer assay, 50 µM B 4 d → 0 µM B (2d)	*DR5:GFP*	Increased	Tao *et al.* (2023)
Signaling	*A. thaliana*	Root TZ and EZ	Transfer assay, 10 μM B 5 d → 0.4 μM B (4d)	*DR5:GUS*	Increased	Martín-Rejano *et al.* (2011)
Signaling	*A. thaliana*	Root tip QC	Seedling assay, 30 μM vs 0.1 μM B (4d, 12d)	*DR5:GFP*	Decreased	Zhou *et al*. (2016)
Signaling	*A. thaliana*	Root MZ and TZ	Transfer assay, 50 µM B 4 d → 0 µM B (2d)	*DII-VENUS*	Decreased	Tao *et al.* (2023)
Signaling	*A. thaliana*	Root TZ, EZ, stele	Seedling assay, 30 μM vs 0.1 μM B (1 week)	*DII-VENUS*	Decreased	Li *et al.* (2015)
Signaling	*A. thaliana*	Root	Shor-term PBA-treatment (10 mM for 35 min)	*DII-VENUS*	Unaltered	Matthes *et al.* (2016)
Signaling	*A. thaliana*	Root EZ and MatZ	Transfer assay,10 μM B 5 d → 0 μM B (4h)	*IAA2:GUS*	Increased	Camacho-Cristóbal *et al.* (2015)
Signaling	*A. thaliana*	Root MZ and EZ	Transfer assay, 10 μM B 5 d → 0 μM B (48h)	*IAA2:GUS*	Decreased	Herrera-Rodríguez *et al.* (2022)
Signaling	*A. thaliana*	Root stele, columella	Transfer assay, 10 μM B 5 d → 0 μM B (48h)	*IAA2:GUS*	Increased	Herrera-Rodríguez *et al.* (2022)
Transport	*A. thaliana*	Root	Transfer assay, 50 µM B 4 d → 0 µM B (2d)	*PIN2/3/4*	Up-regulated	Tao *et al.* (2023)
Transport	*A. thaliana*	Root	Transfer assay,10 μM B 5 d → 0 μM B (4h)	*PIN2*	Down-regulated	Camacho-Cristóbal *et al.* (2015)
Transport	*A. thaliana*	Root tip	Transfer assay, 50 µM B 4 d → 0 µM B (2d)	PIN2-GFP	Increased	Tao *et al.* (2023)
Transport	*A. thaliana*	Root	Short-term PBA-treatment (10 mM for 30 min)	PIN2-GFP	Unaltered localization	Matthes *et al.* (2016)
Transport	*A. thaliana*	Root TZ, EZ, stele	Seedling assay, 30 μM vs 0.1 μM B (1 week)	PIN2-GFP	Unaltered abundance and localization	Li *et al.* (2015)
Transport	*A. thaliana*	Root tip	Transfer assay, 50 µM B 4 d → 0 µM B (2d)	PIN3-GFP, PIN4-GFP	Increased	Tao *et al.* (2023)
Transport	*A. thaliana*	Root	Transfer assay, 50 µM B 4 d → 0 µM B (2d)	*PIN1/7*	Not altered	Tao *et al.* (2023)
Transport	*A. thaliana*	Root tip	Transfer assay, 50 µM B 4 d → 0 µM B (2d)	PIN1-GFP	Not altered	Tao *et al.* (2023)
Transport	*A. thaliana*	Root	Short-term PBA-treatment (10 mM for 30 min)	PIN1-GFP	Altered localization	Matthes *et al.* (2016)
Transport	*A. thaliana*	Whole root, root TZ, EZ, stele	Seedling assay, 30 μM vs 0.1 μM B (1 week)	PIN1-GFP	Decreased	Li *et al.* (2015)
Transport	*A. thaliana*	Root tip QC	Seedling assay, 30 μM or 0.1 μM B (12d)	PIN1-GFP	Decreased	Zhou *et al*. (2016)
Transport	*A. thaliana*	Root tip	Transfer assay, 50 µM B 4 d → 0 µM B (2d)	PIN7-GFP, AUX1-YFP	Not altered	Tao *et al.* (2023)
Transport	*A. thaliana*	Root	Transfer assay, 50 µM B 4 d → 0 µM B (2d)	*AUX1*	Not altered	Tao *et al.* (2023)
Transport	*A. thaliana*	Root	Transfer assay,10 μM B 5 d → 0 μM B (4h)	*AUX1*	Down-regulated	Camacho-Cristóbal *et al.* (2015)
Transport	*A. thaliana*	Root	Transfer assay, 10 μM B 5 d → 0 μM B (48h)	*AUX1*	Down-regulated	Herrera-Rodríguez *et al.* (2022)
Auxin levels	*Z. mays*	Leaves	*tls1* versus WT, soil, 0.08 ppm B	IAA, IAA-Asp	Not altered	Matthes *et al.* (2023)
Conjugation	*Z. mays*	Tassel meristem	*tls1* versus WT, soil, 0.08 ppm B	*aas1/2/12*	Up-regulated	Matthes *et al.* (2022)
Conjugation	*Z. mays*	Tassel meristem	*tls1* versus WT, soil, 0.08 ppm B	*GH3.8*	Up-regulated	Matthes *et al.* (2022)
Signaling	*Z. mays*	Tassel meristem	*tls1* versus WT, soil, 0.08 ppm B	*IAA7/17/26/24*	Up-regulated	Matthes *et al.* (2022)
Transport	*Z. mays*	Tassel meristem	*tls1* versus WT, soil, 0.08 ppm B	*ABCB/PGP*	Down-regulated	Matthes *et al.* (2022)
Transport	*Z. mays*	Shoot apical meristem	*tls1* versus WT, soil, 0.08 ppm B	PIN1a-YFP	Altered	Matthes *et al.*, 2023
Auxin levels	*O. sativa*	Panicles	*asa* versus WT	IAA	Not altered	Zhou *et al.* (2021)

Abbreviations: *AAS*, *AUXIN AMIDO SYNTHETASE*; ABCB/PGP, ATP-binding cassette subfamily B/P-glycoprotein*; ASA, APICAL SPIKELET ABORTION; AUX1*, *AUXIN1*; B, boron; DII, Aux/IAA auxin-interaction domain II; DR5, synthetic auxin-response promoter; EZ, elongation zone; GFP, green fluorescent protein; GH, Gretchen Hagen; GUS, β-glucuronidase; IAA, indole-3-acetic acid; IAA-Asp, IAA-aspartate; IAA-Glu, IAA-glutamic acid; IAAla, indole-3-acetyl-L-alanine; IAA-Me, IAA-methyl ester; MatZ, maturation zone; MZ, meristem zone; *NIT1*, *NITRILASE1*; PBA, phenylboronic acid; *PIN*, *PINFORMED*; QC, quiescent center; *TAA*, *TRYPTOPHAN AMINOTRANSFERASE OF ARABIDOPSIS*; *TAR2*, *TRYPTOPHAN AMINOTRANSFERASE RELATED2*; *tls1*, *tassel-less1*; TZ, transition zone; WT, wild type; YFP, yellow fluorescent protein; *YUC*, *YUCCA*.

**Table 2.** Molecular alterations in the cytokinin-mediated hormone cascade upon boron deficiency (-B) in Arabidopsis (*A. thaliana*) and maize (*Z. mays*).

Cytokinin cascade	Species	Organ/Tissue	Conditions (-B)	Molecular component	Effect (-B)	Literature
Signaling	*A. thaliana*	Root	Transfer assay, 10 μM B 3d → 0 μM B (1d)	*CRE1WOL/AHK4*	Down-regulated	Abreu *et al.* (2014)
Signaling	*A. thaliana*	Root apical meristem	Transfer assay, 30 μM B 5d → 0.03 μM B or 0 μM B (4h, 24h, 48h)	TSCn:GFP	Increased	Poza-Viejo *et al.* (2018)
Signaling	*A. thaliana*	Root vascular cylinder and MZ	Transfer assay, 30 μM B 5d → 0.03 μM B or 0 μM B (5d)	*ARR5:GUS*	Increased	Poza-Viejo *et al.* (2018)
Signaling	*A. thaliana*	Root	Transfer assay, 30 μM B 5d → 0.03 μM B or 0 μM B (4h, 24h, 48h)	*ARR5:GUS*	Increased	Poza-Viejo *et al.* (2018)
Signaling	*A. thaliana*	Root	Transfer assay, 10 µM B 5d → 0 µM B (48h)	*ARR5:GUS*	Increased	Herrera-Rodríguez *et al.* (2022)
Biosynthesis	*Z. mays*	Tassel meristem	*Zmtls1* versus WT, soil, 0.08 ppm B	*log7*	Down-regulated	Matthes *et al.* (2022)
Signaling	*Z. mays*	Tassel meristem	*Zmtls1* versus WT, soil, 0.08 ppm B	TCS:RFP	Decreased	Matthes *et al.* (2022)

Abbreviations: A-ARR, Type-A Arabidopsis RESPONSE REGULATOR; *AHK4*, *ARABIDOPSIS HISTIDINE KINASE4*; *ARR*, *Arabidopsis thaliana Response Regulator*; B, boron; CK, cytokinin; *CRE1*, *CYTOKININ RESPONSE1*; GFP, green fluorescent protein; GUS, β-glucuronidase; *log7*, *lonelyguy7*; MZ, meristem zone; RFP, red fluorescent protein; TCS/TSC(n), two-component system; *WOL*, *WOODENLEG*.

#### Boron deficiency-induced alterations of the auxin cascade

The biological activity of indole-3-acetic acid (IAA), the major natural auxin in plants, is influenced by its biosynthesis, signaling, and, particularly, its directional cell-to-cell transport (polar auxin transport) (as reviewed in Adamowski and Friml, 2015; Casanova-Sáez *et al.*, 2021; Pernisová and Vernoux, 2021). Boron deficiency has been shown to affect auxin biosynthesis, levels, transport, and signaling across different plant species in a tissue-, time-, and genotype-dependent manner (Table 1; Supplementary Table S1). Although early studies suggested a potential role of boron in auxin biosynthesis (Eaton, 1940; Dyar and Webb, 1961), more recent evidence highlighted that the mechanisms underlying boron deficiency-induced alterations of auxin signaling and levels are complex and not limited to altered auxin biosynthesis (Martín-Rejano *et al.*, 2011; Camacho-Cristóbal *et al.*, 2015; Li *et al.*, 2015, 2016; Herrera-Rodríguez *et al.*, 2022; Matthes *et al.*, 2022).

In Arabidopsis roots, boron deficiency induced an increase in auxin signaling which contributed to the boron deficiency-induced inhibition of primary root elongation (Martín-Rejano *et al.*, 2011; Camacho-Cristóbal *et al.*, 2015; Li *et al.*, 2015; Herrera-Rodríguez *et al.*, 2022). The activity of the auxin signaling marker *IAA2:GUS* increased in the stele and the cortex of the root elongation and maturation zones, whereas it either increased or decreased in the epidermis of the root elongation and maturation zones (Camacho-Cristóbal *et al.*, 2015; Herrera-Rodríguez *et al.*, 2022) (Table 1). Likewise, an increase in auxin signaling was reported in the meristem zone, the transition zone, and the elongation zone of boron-deficient roots and in boron-deficient shoot tissue (Martín-Rejano *et al.*, 2011; Li *et al.*, 2015; Tao *et al.*, 2023). A decrease of auxin signaling, however, was reported in cotyledons or the root QC (Matthes and Torres-Ruiz, 2016; Zhou *et al.*, 2016). Furthermore, IAA levels increased in boron-deficient compared with boron-sufficient Arabidopsis roots (Tao *et al.*, 2023). Boron deprivation in the root, however, did not influence the expression of auxin biosynthesis genes, including *TRYPTOPHAN AMINOTRANSFERASE OF ARABIDOPSIS1* (*TAA1*), *YUCCA3* (*YUC3*), *YUC9* and *NITRILASE1* (*NIT1*). In contrast, in Arabidopsis shoots, the expression levels of *TAA1*, *YUC3*, *YUC9*, and *NIT1* were up-regulated under boron-deficient conditions (Tao *et al.*, 2023), highlighting tissue specific differences in the molecular pathways affected by boron deficiency (Table 1).

In maize and rice boron transporter mutants, IAA levels were not altered in developing leaves (in *tls1* mutants) or in panicles (in *asa* mutants) compared with the respective wild-type siblings (Table 1) (Zhou *et al.*, 2021; Matthes *et al.*, 2023). An enhancement of the boron deficiency-induced leaf defects, however, was observed in double mutants of *tls1* with the auxin biosynthesis mutant, *vanishing tassel2* (Phillips *et al.*, 2011; Matthes *et al.*, 2023). This enhancement of boron deficiency-induced defects by lowering auxin levels was not observed in tassel meristems, although RNA-sequencing detected enhanced expression of a few auxin inactivation genes and auxin responsive genes in *tls1* tassel meristems compared with wild-type siblings (Matthes *et al.*, 2022, 2023). This indicates the tissue-specific importance of auxin levels in the boron deficiency response of maize.

In oilseed rape, low-boron conditions affected the expression of auxin biosynthesis genes and auxin levels in a tissue-, time- and genotype-dependent manner (Supplementary Table S1; Zhou *et al.*, 2016; Eggert and von Wirén, 2017; Pommerrenig *et al.*, 2022), suggesting auxin metabolism is related to the boron utilization efficiency in this plant species. Prolonged boron deficiency (20 days) reduced both IAA levels and the expression of the auxin biosynthesis gene *NIT1* in the boron-inefficient cultivar W10 in all analyzed root and shoot tissues (Zhou *et al.*, 2016). In the boron-efficient cultivar QY10, however, IAA levels remained unaltered or decreased in a tissue-dependent manner (Zhou *et al.*, 2016) (Supplementary Table S1). In contrast, accumulation of IAA, indole-3-acetonitrile, and indole-3-acetamide was reported with progressing boron deficiency in 7-day-old shoots of the varieties Campino and Alpaga (Eggert and von Wirén, 2017) and in the boron-inefficient oilseed rape accession CR2262 compared with the boron-efficient cultivar CR2267 (Pommerrenig *et al.*, 2022). In addition, levels of the convertible storage form IAA-methyl ester were higher in CR2262 compared with CR2267 under boron-deficient conditions (Pommerrenig *et al.*, 2022). These deviating findings highlight the importance of genotypic differences, which can lead to contrasting molecular responses induced by boron deficiency.

Fine tuning of auxin concentrations, primarily directed by an alteration of polar auxin transport processes, is crucial for root and shoot growth under boron-deficient conditions in various plant species (Martín-Rejano *et al.*, 2011; Camacho-Cristóbal *et al.*, 2015; Li *et al.*, 2015, 2016; Matthes and Torres-Ruiz, 2016; Zhou *et al.*, 2016; Herrera-Rodríguez *et al.*, 2022; Matthes *et al.*, 2023; Tao *et al.*, 2023). For example, in Arabidopsis, individual auxin transporters differentially contributed to the boron deficiency-induced phenotypic defects in a tissue-dependent manner, further dependent on the severity of boron deficiency (Table 1). Shootward auxin transport via PINFORMED2/ETHYLENE-INSENSITIVE ROOT1 (PIN2/EIR1) was particularly enhanced in root tissues, whereas rootward auxin transport via AUXIN1 (AUX1) and PIN1 was reduced under boron-deficient conditions. These alterations were consequently shown to contribute to the boron deficiency-induced inhibition of root cell elongation in Arabidopsis (Martín-Rejano *et al.*, 2011; Camacho-Cristóbal *et al.*, 2015; Li *et al.*, 2015; Zhou *et al.*, 2016; Herrera-Rodríguez *et al.*, 2022; Tao *et al.*, 2023). Moreover, boron deficiency-induced alterations of PIN1-mediated auxin transport has emerged as species-independent early molecular response upon boron deficiency (Li *et al.*, 2015, 2016; Zhou *et al.*, 2016; Matthes *et al.*, 2023; Tao *et al.*, 2023).

Dependent on the severity of boron deficiency, transcript levels of *PIN2* either increased (no boron) or decreased (low boron) in shoots and roots (Camacho-Cristóbal *et al.*, 2015; Tao *et al.*, 2023). Furthermore, *PIN3* transcripts were up-regulated in boron-deficient roots and shoots, whereas *PIN4* levels were only up-regulated in boron-deficient roots (Tao *et al.*, 2023). Consequently, Tao *et al.*, 2023 showed an increase in PIN2:GFP, PIN3:GFP, and PIN4:GFP levels under boron-deficient conditions, although no differences in the abundance or subcellular localization of PIN2:GFP were detected in roots between low and sufficient-boron conditions (Li *et al.,* 2015). Similarly, different studies reported contrasting root responses of *pin2* mutants to boron deficiency. The ethylene-insensitive mutant *pin2*/*eir1-4* was less affected by boron deficiency (Camacho-Cristóbal *et al.*, 2015), whereas the *pin2/eir1-1* mutant was more sensitive to low boron conditions compared with the wild type and exhibited a stronger reduction in meristem size (Li *et al.*, 2015). Enhanced root growth was further reported in the *pin2* and *pin3* mutants alongside reduced auxin levels under boron-deficient conditions (Tao *et al.*, 2023). These deviating findings might depict tissue-, cell-type-, and genotype-specific involvements of PIN2 under boron-deficient conditions, which warrants systematic investigation.

Mutants in the auxin efflux transporter genes *PIN1* and *PIN7* developed shorter primary roots under boron deprivation compared with the wild-type control. The expression of *PIN1* and *PIN7* and the abundance of PIN1:GFP and PIN7:GFP were not altered (Tao *et al.*, 2023), although additional studies report reduced PIN1:GFP signals under low boron conditions in roots (Li *et al.*, 2015; Zhou *et al.*, 2016). On a cellular level, reduced localization of PIN1:GFP at the plasma membrane was reported in cotyledon cells of Arabidopsis embryos and seedling roots treated with the putative boron deficiency mimic PBA (Matthes and Torres-Ruiz, 2016), which was proposed to be causal for altered cell division of the hypophysis.

The auxin influx transporter AUX1, appears to be mechanistically involved in the boron deficiency-induced primary root defects in Arabidopsis (Camacho-Cristóbal *et al.*, 2015; Herrera-Rodríguez *et al.*, 2022; Tao *et al.*, 2023). *AUX1* expression was down-regulated under boron-deficient conditions, which correlated with tissue-specific alterations of auxin signaling, as monitored with the *IAA:GUS* marker, in the meristematic zone, elongation zone, and stele (Table 1; Camacho-Cristóbal *et al.*, 2015; Herrera-Rodríguez *et al.*, 2022).

PIN1a:YFP showed alterations in distribution, accumulation and localization in the shoot apical meristem and developing tassel meristem of the maize boron transporter mutant *tls1* (Matthes *et al.*, 2023), suggesting reduced PIN1a-mediated auxin transport in the boron-deficient maize shoot. In oilseed rape, long term boron deficiency (20 days) led to decreased expression of *PIN1* in roots of boron-efficient and boron-inefficient cultivars. *PIN2* expression, however, was only affected in shoot tissue. Short term boron deficiency (up to 12 h) led to an increase of *PIN1* expression in root tissue only in the boron-inefficient cultivar (Supplementary Table S1; Zhou *et al.*, 2016).

#### Boron deficiency-induced alterations of the ethylene cascade

Several studies have shown that boron deficiency induces an accumulation of ethylene levels, which consequently increased ethylene signaling in the Arabidopsis root. This involvement of the ethylene cascade mechanistically contributed to boron deficiency-induced primary root elongation defects and acts upstream of the auxin cascade (Martín-Rejano *et al.*, 2011; Camacho-Cristóbal *et al.*, 2015; Herrera-Rodríguez *et al.*, 2022). Under boron-deficient conditions, the transcriptional levels of the ethylene biosynthesis gene *1-AMINOCYCLOPROPANE-1-CARBOXYLIC ACID* (*ACC*) *SYNTHASE11* (*ACS11)* were up-regulated in Arabidopsis roots and the ethylene reporter *EBS:GUS* was induced (Martín-Rejano *et al.*, 2011; Camacho-Cristóbal *et al.*, 2015; Herrera-Rodríguez *et al.*, 2022). Conversely, blocking ethylene signaling or inhibiting ethylene biosynthesis suppressed the inhibition of cell elongation caused by boron deficiency, likely by restoring the boron deficiency-induced defects on auxin signaling. Moreover, ethylene insensitive mutants and ethylene receptor loss-of-function mutants were less sensitive to boron deficiency (Martín-Rejano *et al.*, 2011; Camacho-Cristóbal *et al.*, 2015; Huang *et al.*, 2021). From a mechanistic point of view, altered ethylene levels and signaling might directly feedback into boron transport. The *pNIP5;1:GUS* reporter showed that the expression of *NIP5;1* was induced by the ethylene precursor ACC throughout the root apical meristem, whereas an ethylene synthesis inhibitor induced *NIP5;1* expression in the maturation zone exclusively in plants transferred to boron-deficient conditions (Gómez-Soto *et al.*, 2019).

#### Boron deficiency-induced alterations of the cytokinin cascade

Cytokinin is emerging as pivotal plant hormone in the boron deficiency response of many plant species (Abreu *et al.*, 2014; Eggert and von Wirén, 2017; Poza-Viejo *et al.*, 2018; Matthes *et al.*, 2022; Pommerrenig *et al.*, 2022). Interactions of boron with the cytokinin cascade have been reported on the level of biosynthesis and signaling (Table 2; Supplementary Table S2). For details about the cytokinin cascade see Argueso and Kieber (2024).

Recent evidence suggests that the boron deficiency-induced reduction of primary root elongation in Arabidopsis is caused by altered cell proliferation in the meristem region, mediated by enhanced cytokinin signaling and levels (Herrera-Rodríguez *et al.*, 2022). Cytokinin signaling was particularly enhanced in the stele and the root apical meristem, as deduced from the increase in cytokinin-inducible markers *Arabidopsis Response Regulator* (*ARR)5*:GUS and *Two Component signaling Sensor* (TCSn):GFP under boron-deficient conditions (Poza-Viejo *et al.*, 2018; Herrera-Rodríguez *et al.*, 2022). The boron deficiency-induced accumulation of cytokinin was shown to inhibit *AUX1* expression and to induce ethylene biosynthesis through *ACS11* (Herrera-Rodríguez *et al.*, 2022), therefore placing cytokinin upstream of ethylene and auxin in the boron deficiency response of the Arabidopsis root. Additionally, the cytokinin receptor gene *CYTOKININ RESPONSE* (*CRE*)*/WOODEN LEG* (*WOL*)*/ARABIDOPSIS HISTIDINE KINASE4* (*AHK4*) was down-regulated under boron-deficient conditions in the root meristem (Abreu *et al.*, 2014). Furthermore, the boron deficiency-induced primary root elongation defects were alleviated in the *ahk4* mutant (Herrera-Rodríguez *et al.*, 2022). Similarly, mutants affected in cytokinin perception and signaling showed increased meristem sizes under low boron conditions compared with the wild type (Poza-Viejo *et al.*, 2018). However, treatment of boron-deficient wild-type roots with the active cytokinin *trans*-zeatin (*t*Z), led to an increase of root meristem size by promoting cell division under severe boron deficiency (Poza-Viejo *et al.*, 2018) and to partial suppression of boron deficiency-induced primary root elongation defects. Moreover, *AtNIP5;1* expression was significantly enhanced in transfer experiments from control to boron-deficient conditions when cytokinin signaling was chemically inhibited (Gómez-Soto *et al.*, 2019). This observation suggested feedback mechanisms onto boron transport. Elevated boron concentrations, however, were not detected. These findings show that boron deficiency-induced alterations of the cytokinin cascade in Arabidopsis are highly complex and tissue-specific. Their full mechanistic understanding warrants future investigations.

In contrast to Arabidopsis, boron deficiency led to reduced cytokinin signaling in the maize tassel meristem of the *tls1* mutant, as indicated by reduced TCS:RFP signals (Matthes *et al.*, 2022). Consequently, enhancing the cytokinin response in the *tls1* mutant partially suppressed its tassel phenotype (absence of a tassel), demonstrated by genetic interaction studies of *tls1* with the meristem pathway gene, *thick tassel dwarf1*, and the cytokinin signaling mutant *aberrant phyllotaxy1* (Giulini *et al.*, 2004; Matthes *et al.*, 2022). Among the differentially expressed genes in tassel meristems of *tls1* was *lonelyguy7 (log7*; Matthes *et al.*, 2022). The cytokinin biosynthesis gene *log7* was down-regulated in *tls1* compared with wild-type siblings, potentially mediating a reduction in cytokinin levels in *tls1* meristems. These observations exemplify a potentially mechanistic involvement of the cytokinin cascade in the boron deficiency response of the maize tassel meristem

In oilseed rape, boron-dependent growth responses were closely associated with *de-novo* synthesis of cytokinins and the re-routing of inactive cytokinin forms towards active forms in a genotype-dependent manner (Supplementary Table S2; Eggert and von Wirén, 2017; Pommerrenig *et al.*, 2022). The levels of the active cytokinins N6-isopentenyladenine (IP) and *cis*-zeatin (*c*Z), and their respective inactive precursors isopentenyl riboside (IPR) and *cis*-zeatin riboside (*c*ZR) dropped drastically under limiting boron supply during early seedling development of two boron-inefficient cultivars. (Eggert and von Wirén, 2017). In contrast, the levels of the precursors IPR, *c*ZR, and *t*ZR highly accumulated in a boron-efficient cultivar compared with a boron-inefficient cultivar during the outgrowth of the shoot apical meristem and emergence of the first leaves under boron-deficient conditions. Likewise, deactivated storage forms of cytokinin accumulated only in the boron-inefficient cultivar under boron-deficient conditions (Supplemetary Table S2; Pommerrenig *et al.*, 2022). Active cytokinins, therefore, were proposed to be shoot growth-promoting signals under low boron conditions in oilseed rape (Pommerrenig *et al.*, 2022).

### Cell wall (in-)dependence of the boron deficiency-induced molecular defects

Boron, in the form of boric acid or the borate anion, can readily complex with diols and polyols. The most stable borate diesters are formed with *cis*-diols of the pentoses, ribose and apiose, the latter being a prominent component of the plant primary cell wall (Rengel *et al.*, 2022). The best characterised function of boron in plants is its complexation with apiosyl residues of the pectic component Rhamnogalacturonan (RG)-II in the cell wall (Kobayashi *et al.*, 1996; Matoh *et al.*, 1996; O’Neill *et al.*, 1996). Boron crosslinks two RG-II-monomers to form a RG-II dimer. The pectin amounts in primary cell walls have, therefore, been directly linked to the boron requirements of plants (Hu *et al.*, 1996). Consequently, differences in cell wall pectin provide explanation for the discrepancies in observed boron deficiency-induced phenotypes between different plant species.

Pectin plays crucial mechanical roles in the primary cell wall and alterations of its composition, specific modifications, and disturbances of the borate-RG-II dimers were shown to influence primary cell wall mechanical properties which are instrumental for normal plant growth, including meristem development (Iwai *et al.*, 2006; Peaucelle *et al.*, 2012; Banwarth-Kuhn *et al.*, 2022). Defects in RG-II crosslinking or side chain composition, in particular, have been associated with the cell elongation phenotypes observed under boron-deficient conditions and include reduced growth, swollen cells walls, and reduced wall tensile strength (O’Neill *et al.*, 2001; Liu *et al.*, 2011; Voxeur *et al.*, 2011; Peng *et al.*, 2021).

Understanding the effects of altered mechanical forces on plant morphogenesis, including possible interactions with known molecular pathways, is being actively researched (as reviewed in Jonsson *et al.*, 2022). Of the molecular pathways affected by boron deficiency, particularly auxin and ethylene appear to be responsive to the mechanical status of a cell. Specifically, cell wall strength and auxin concentration were proposed to be causally related in a feedback loop (Aryal *et al.*, 2020) and PIN1 localization was shown to depend on mechanical forces (Heisler *et al.*, 2010; Feraru *et al.*, 2011). Moreover, to maintaining proper size and shape of the shoot apical meristem, not only the meristem maintenance factor WUS and cytokinin are decisive, but also cell wall-mediated mechanical cues in a layer-specific manner (Banwarth-Kuhn *et al.*, 2022).

These findings suggest that the boron deficiency-induced alterations of the discussed molecular pathways in plant meristems might be secondary to cell wall defects. Additionally, there are several observations that provoke speculations about cell wall-independent functions of boron. The *murus1* (*mur1*) mutant in Arabidopsis, which is deficient in fucose, the main sugar found in RG-II, showed varying degrees of dwarfism, yet no reproductive defects were reported (O’Neill *et al.*, 2001). In addition, cell division, cell elongation, and root meristem exhaustion defects were reported to occur in parallel shortly after boron deprivation (Poza-Viejo *et al.*, 2018) and molecular alterations were reported prior any visible cell wall defects (Matthes *et al.*, 2022). Moreover, boron was shown to be beneficial for the development of animals, which do not have a cell wall (Reguera *et al.*, 2019). Recent progress further identified the Golgi-apparatus as the subcellular site of boron-bridging of RG-II (Begum and Fry, 2022), which exemplified new cellular locations for boron functions outside the cell wall. Furthermore, boron-binding proteins were identified in the plasma membrane (Wimmer *et al.*, 2009; Voxeur and Fry, 2014), which places the plasma membrane as an integration spot of biochemical and mechanical cues, potentially exerting a pivotal function in mediating boron deficiency defects.

## Conclusions and outlook

**Fig. 1.**
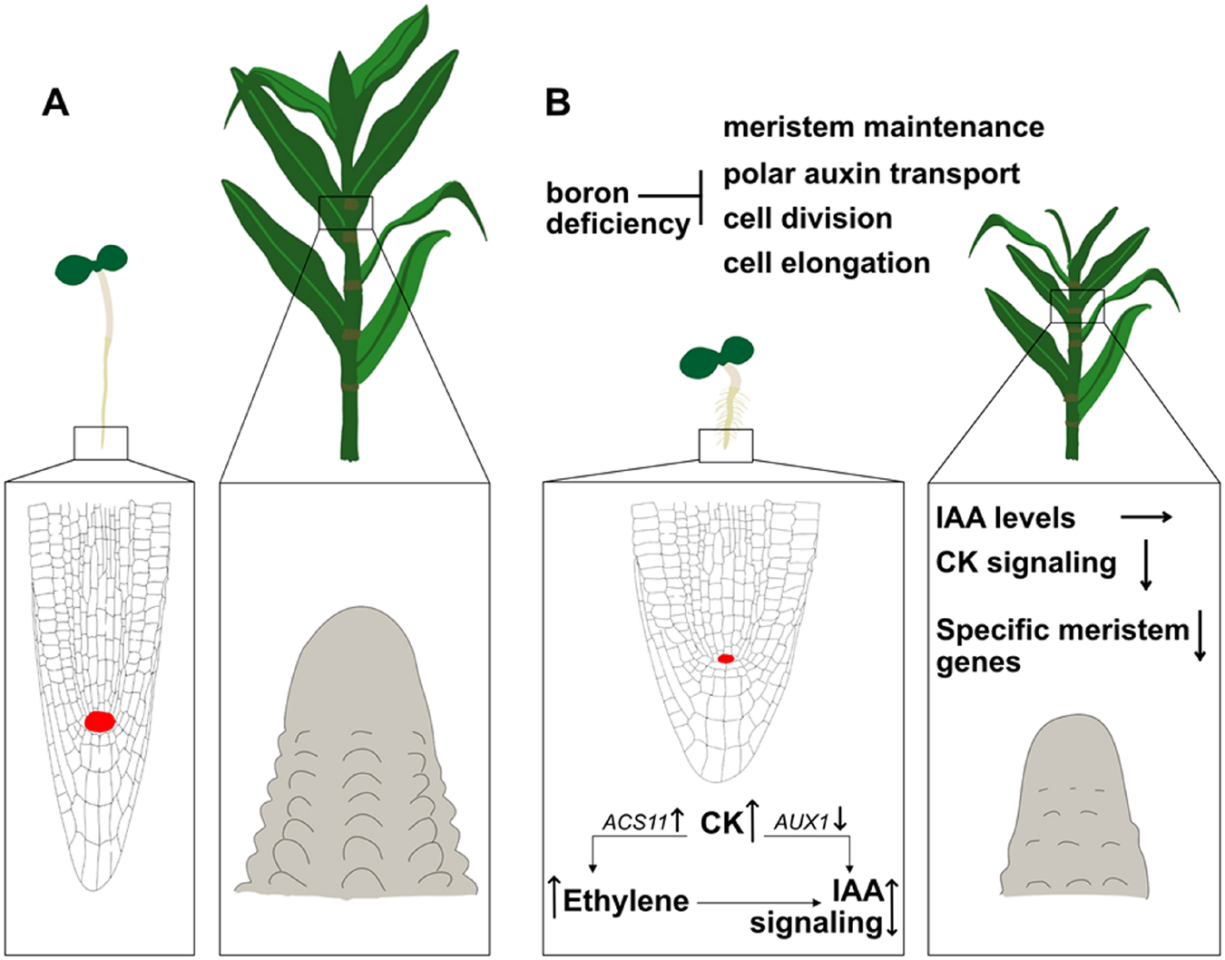
Differences and similarities of boron deficiency-induced molecular defects in the Arabidopsis (*Arabidopsis thaliana*) root and the maize (*Zea mays*) tassel meristems. (A) Under normal boron conditions, regular meristem developmental programs lead to normal growth of Arabidopsis and maize. (B) Under boron-deficient conditions, meristem maintenance, cell division, and cell elongation are inhibited in the Arabidopsis root meristem and the maize tassel meristem. Specifically, inhibition of polar auxin transport is an early detectable defect in both meristems. In the Arabidopsis root meristem, cytokinin (CK) signaling and levels are increased, which lead to altered auxin (IAA) signaling. Both enhancement and decrease of IAA signaling had been reported and correlated with altered expression of the auxin transport gene *AUXIN1* (*AUX1*). These molecular defects lead to a reduction in root apical meristem size (red), which mediates the decrease in primary root elongation in the boron-deficient Arabidopsis seedling. Enhanced ethylene signaling through up-regulation of the ethylene biosynthesis gene *1-AMINOCYCLOPROPANE-1-CARBOXYLIC ACID SYNTHASE 11* (*ACS11*) was shown to be an early molecular defect in the boron-deficient Arabidopsis root, which in turn contributes to altered IAA signaling. Furthermore, a thick and stunted primary root tip and an increase in root hairs is observed. In contrast, in the maize tassel meristem, IAA levels are unaltered and CK signaling is reduced. In addition, boron deficiency leads to a down-regulation of the expression of specific meristem genes. Consequently, the growth of the tassel meristem is halted, leading to a reduction in tassel size under boron-deficient conditions.

In this review we highlighted the importance of the micronutrient boron for meristem developmental processes. The hormones auxin, cytokinin, and ethylene appear as intriguing factors, shaping the observed boron deficiency-induced defects in meristems. Active cytokinins emerge as master regulators for shoot meristem maintenance under boron-deficient conditions in many species (Eggert and von Wirén, 2017; Matthes *et al.*, 2022; Pommerrenig *et al.*, 2022; Chen *et al.*, 2024). They also emerge as important regulators for Arabidopsis root meristem development under boron-deficient conditions acting upstream of auxin and ethylene (Abreu *et al.*, 2014; Poza-Viejo *et al.*, 2018; Herrera-Rodríguez *et al.*, 2022). Despite auxin being considered the main regulator of root growth under boron-deficient conditions in Arabidopsis (Chen *et al.*, 2022), global alterations of auxin levels in boron-deficient shoot meristems, appear to be less consequential for boron deficiency-induced shoot meristem defects (Zhou *et al.*, 2021; Matthes, 2022). Enhanced auxin signaling, mediated by ethylene- and cytokinin-dependent pathways, however, is instrumental in the Arabidopsis boron deficiency-induced root meristem defects (Fig. 1) (Martín-Rejano *et al.*, 2011; Camacho-Cristóbal *et al.*, 2015; Poza-Viejo *et al.*, 2018; Herrera-Rodríguez *et al.*, 2022). Moreover, evidence for a boron deficiency-induced inhibition of polar auxin transport manifests as one of the early detectable alterations in different plant species and different meristem types (Li *et al.*, 2015; Matthes and Torres-Ruiz, 2016; Matthes *et al.*, 2023; Tao *et al.*, 2023).

It is, nevertheless, evident that various hormone cascades or interactions and additional molecular processes, including cell wall signaling pathways, further contribute to the boron deficiency-induced defects in plant meristems. The finding that the expression of different transcription factor families is affected by boron levels, including those that are directly involved in meristem development processes, is particularly interesting (Matthes *et al.*, 2022; Verwaaijen *et al.*, 2023; Chen *et al.*, 2024). They provide direct targets for how boron might integrate into the plant developmental program and how the boron deficiency-induced signal might be transduced (González-Fontes *et al.*, 2013). Moreover, recent evidence linked the oilseed rape flowering-without-seed-setting phenotype with the activity of metacaspases, which are involved in programmed cell death (Verwaaijen *et al.*, 2023) and in maize a non-boron transporter related gene was shown to be correlated with boron homeostasis (Chu *et al.*, 2025). These recent findings showcase the diversity and topicality of current boron research. Regarding the characterized function of boron in crosslinking RG-II in the cell wall, it remains pivotal to dissect how the boron deficiency-induced molecular alterations can be functionally linked with a disturbed cell wall. To deduce primary versus secondary molecular functions of boron, it will be important to combine cell- and layer-specific studies with mechanical and molecular analyses. To do so, particularly in plant meristems, will remain to be challenging.

## Supplementary data

The following supplementary data are available at *JXB* online.

Table S1. Molecular alterations in the auxin-mediated hormone cascade upon boron deficiency in *Brassica napus*.

Table S2. Molecular alterations in the cytokinin-mediated hormone cascade upon boron deficiency in *Brassica napus*.

eraf036_suppl_Supplementary_Tables_S1-S2
